# Real‐world assessment of comprehensive genome profiling impact on clinical outcomes: A single‐institution study in Japan

**DOI:** 10.1002/cam4.70249

**Published:** 2024-09-24

**Authors:** Kei Kunimasa, Naotoshi Sugimoto, Tomoyuki Yamasaki, Yoji Kukita, Fumie Fujisawa, Tazuko Inoue, Yuko Yamaguchi, Mitsuko Kitasaka, Daisuke Sakai, Keiichiro Honma, Toru Wakamatsu, Sachiko Yamamoto, Takuji Hayashi, Seiji Mabuchi, Jun Okuno, Takahisa Kawamura, Yugo Kai, Makiko Urabe, Kazuo Nishimura

**Affiliations:** ^1^ Department of Thoracic Oncology Osaka International Cancer Institute Osaka Japan; ^2^ Department of Genetic Oncology Osaka International Cancer Institute Osaka Japan; ^3^ Department of Medical Oncology Osaka International Cancer Institute Osaka Japan; ^4^ Department of Endocrinology and Metabolism, Clinical Laboratory Osaka International Cancer Institute Osaka Japan; ^5^ Laboratory of Genomic Pathology Osaka International Cancer Institute Osaka Japan; ^6^ Department of Medical Oncology Shiga General Hospital Shiga Japan; ^7^ Department of Diagnostic Pathology and Cytology Osaka International Cancer Institute Osaka Japan; ^8^ Musculoskeletal Oncology Service Osaka International Cancer Institute Osaka Japan; ^9^ Department of Gastrointestinal Oncology Osaka International Cancer Institute Osaka Japan; ^10^ Department of Urology Osaka International Cancer Institute Osaka Japan; ^11^ Department of Gynecology Osaka International Cancer Institute Osaka Japan; ^12^ Department of Breast and Endocrine Surgery Osaka International Cancer Institute Osaka Japan; ^13^ Department of Hepatobiliary and Pancreatic Oncology Osaka International Cancer Institute Osaka Japan

**Keywords:** clinical trial, comprehensive genomic profiling, genomically matched therapy, real world data, solid tumor

## Abstract

**Introduction:**

Comprehensive genome profiling (CGP) has revolutionized healthcare by offering personalized medicine opportunities. However, its real‐world utility and impact remain incompletely understood. This study examined the extent to which CGP leads to genomically matched therapy and its effectiveness.

**Methods:**

We analyzed data from advanced solid tumor patients who underwent CGP panel between December 2019 and May 2023 at the Osaka International Cancer Institute. Patient demographics, specimen details, and expert panel assessments were collected. Turnaround time (TAT) and genomically matched therapy outcomes were analyzed. Gene alterations and their co‐occurrence patterns were also assessed.

**Results:**

Among 1437 patients, 1096 results were available for analysis. The median TAT was 63 [28–182] days. There were 667 (60.9%) cases wherein recommended clinical trials were presented and there were 12 (1.1%) cases that could be enrolled in the trial and 25 (2.3%) cases that could lead to therapies under insurance reimbursement. The median progression free survival of the trial treatment was 1.58 months (95% CI: 0.66–4.37) in clinical trials and 3.66 months (95% CI: 2.14–7.13) in treatment under insurance. Pathologic germline variants were confirmed in 15 patients (1.3%). Co‐alteration of *CDKN2A*, *CDKN2B*, and *MTAP* was significantly observed in overall population.

**Conclusion:**

The effectiveness of the genomically matched therapy based on the CGP panel was unsatisfactory. Expansion of clinical trials and utilization of remote clinical trials are required to ensure that the results of the CGP panel can be fully returned to patients.

## Graphical abstract

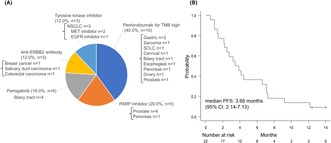

## 1 INTRODUCTION

Genomics has emerged as a transformative field in healthcare, offering new opportunities for personalized medicine.^1^, ^2^, ^3^ Comprehensive genome profiling (CGP) is a powerful tool for deciphering an individual's genetic makeup that holds immense promise for guiding clinical decision‐making.^4^, ^5^, ^6^ The application of genomic testing in clinical practice has rapidly expanded with advancements in technology and decreasing costs.^7^, ^8^ However, the true utility and impact of comprehensive genome profiling in real‐world settings remain to be fully understood. Previous studies have demonstrated the potential benefits of genomic testing in clinical practice.^9^, ^10^, ^11^ For instance, comprehensive genome profiling identified clinically actionable alterations in more than 30% of patients with advanced cancers, leading to tailored treatment approaches.^12^, ^13^, ^14^ Moreover, a systematic review highlighted the positive impact of genomic testing on diagnostic yield and treatment selection in various genetic disorders.^15^, ^16^ These studies provide important insights into the potential of genomic testing but are limited in their scope and generalizability.

Despite the existing evidence,^17^, ^18^, ^19^ several gaps remain in our understanding of the utility and usage of comprehensive genome profiling in clinical practice. First, large‐scale observational studies that capture real‐world data and patient outcomes are lacking. Second, the specific impact of genomic testing in a single institution in Japan where genetic diversity and clinical practices may differ from Western populations remains largely unexplored. Considering these knowledge gaps, the objective of this study was to assess the impact of comprehensive genome profiling in clinical practice within a single institution in Japan. We aimed to provide robust evidence on the utility and usage of genomic testing by leveraging a large dataset spanning a diverse patient population and a significant timeframe.

It is crucial to evaluate the real‐world utility and the impact of comprehensive genome profiling as genomic medicine continues to advance. This study aims to contribute to the broader understanding of personalized medicine by assessing the impact of genomic testing in a single institution in Japan. The results presented in this study will have implications for clinical practice, policy‐making, and the delivery of precision healthcare.

## 2 PATIENTS AND METHODS

### 2.1 Study participants and CGP panel analysis methodology

This research incorporated individuals diagnosed with advanced solid malignancies who were subjected to CGP panel examinations at the Osaka International Cancer Institute from December 2019, the time when the CGP panel received insurance coverage approval in Japan, until May 2023. Data regarding the patients' demographic details, such as age and sex, along with their specific type of cancer and the treatment history up to the point of undergoing the CGP panel assessment, were systematically gathered. Prior to the submission of tests, each study participant was queried about their preference for sharing the outcomes of the CGP panel analysis with entities beyond themselves, including any data concerning genetic cancer risks. For cases involving tumor samples, the choice between conducting a Foundation One (F1) panel or an OncoGuide NCC oncopanel (NCC panel) rested with the treating physician. In instances where tissue samples were employed, the eligibility for submitting these samples for CGP panel evaluation was determined by a pathologist. This determination was based on criteria such as the size of the tumor area, the proportion of tumor cells present, and the duration for which the specimen had been stored, following the acquisition of informed consent from the patients. We also examined the proportion of cases where specimens were deemed unsuitable for submission based on the pathologist's assessment, and whether these individuals were subject to further diagnostic evaluations. Starting from August 2021, with the introduction of F1 liquid, an alternative approach was offered to those without available tissue samples; consenting individuals underwent CGP panel testing employing F1 liquid.

### 2.2 CGP Panel Expert Review Process

Within the Osaka International Cancer Institute, a specialized team reviewed the findings from CGP panel tests and provided explanations to the patients, adhering to the methods outlined in previous literature.^20^, ^21^ This multidisciplinary team included a dedicated oncologist for each type of organ involved, alongside a clinical geneticist, genetic counseling specialist, pathologist, clinical study coordinator, and pharmacist.^22^ Initially, the team assessed the reports to verify the DNA quality, ensuring it met the criteria for analysis or was deemed acceptable, and verified the status of microsatellite instability (MSI) and tumor mutation burden (TMB). For identified genetic variations, the potential for oncogenesis was determined referencing the specific gene panel reports and the guidelines from the Center for Cancer Genomics and Advanced Therapeutics (C‐CAT).^23^ Recommendations for treatment targeting oncogenic mutations were made based on clinical trial outcomes, predominantly from studies conducted in Japan, and categorized according to C‐CAT's recommendation scale ranging from A to F.^24^, ^25^ The option of medication therapy via the patient offer system was additionally presented. Genetic variations potentially linked to hereditary cancers were treated as incidental findings. The criteria for determining this were in accordance with the standards of the American College of Medical Genetics and Genomics (ACMG).^26^ In cases where such findings were revealed, we contemplated recommending genetic counseling for the patient. Every deliberation took into account the patient's unique medical background, emphasizing their prior treatment experiences.

### 2.3 Turnaround time (TAT)

The TAT (days) included the time needed for the evaluation of tumor cell content of stored formalin‐fixed, paraffin‐embedded (FFPE) specimens by a pathologist after the order, cutting FFPE specimens, submission of the cut specimens to the laboratory, analysis by the expert panel with the report returned by the laboratory, and explanation of the results by the expert panel to the patient.

### 2.4 Genomically matched therapy

Expert panel results showed that the drug treatment for detected gene alterations was genomically matched. The data were separately collected for treatment in clinical trials and treatment under insurance reimbursement. In the case of clinical trials, the drug name was unspecified, and the number of days from the time of entry into the clinical trial until death or survival was counted. In the case of treatment under the insurance scheme, the number of days from the time of treatment introduction to death or survival was counted as well as the drug name.

### 2.5 Statistical analysis

The Kruskal–Walli's test was used to compare TMB by cancer type after confirming the normality of the data. The Bonferroni method was used for multiple comparisons and the significance level was set at 5%. Mutual exclusivity and co‐occurrence of gene mutations were assessed using Fisher's exact test. Statistical analyses were performed using the EZR software version 1.29 (Saitama Medical Center, Jichi Medical University, Saitama, Japan).^27^ The top 20 most frequently mutated genes from each tumor type were selected for the assessment. The intensity of association was scored by −1 × log10(*p*‐value). Heatmap figures were depicted by the ggplot2 library of R program.^28^ Survival curves were generated using the Kaplan–Meier method and compared using the Log rank test.

## 3 RESULTS

### 3.1 Patient characteristics

**FIGURE 1:**
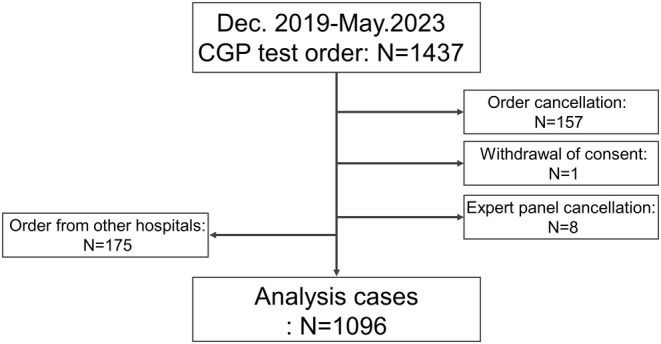
Patient flow chart. Flow chart of 1437 patients who consented to CGP panel evaluation.

**FIGURE 2:**
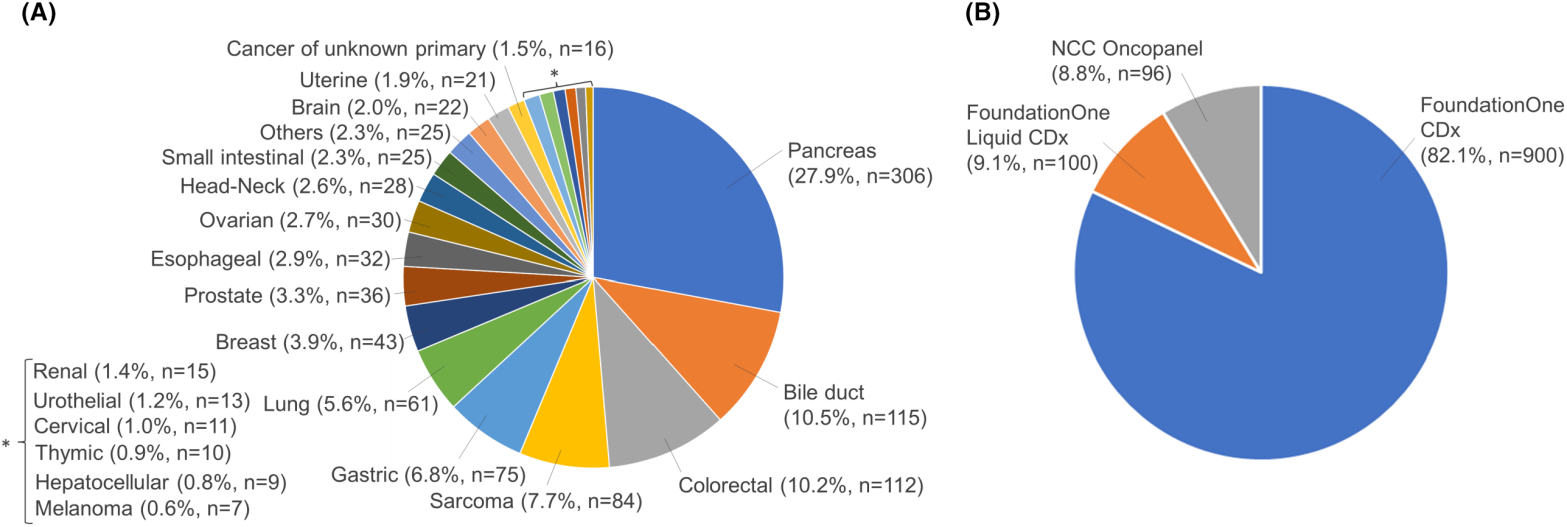
Number of samples that underwent CGP panel by cancer type (A) and used CGP panel (B).

During the study period, 1437 patients consented to CGP panel testing. Of these, 157 patients (10.9%) had their tests canceled owing to specimen failure, one patient withdrew consent after ordering the test, 8 patients (0.5%) had their expert panels canceled owing to the death of the patient who submitted the test, and 175 patients (12.2%) were referred from other hospitals and were excluded since their consent was not obtained for this study. Finally, 1096 results (76.3%) were available for analysis (Figure 1). The median age for all patients was 63 [16–88] years, with 588 (53.6%) patients being men and 508 (46.4%) women. The submitted cancer types are shown in Figure 2A. The top 10 cancer types were: (1) pancreatic cancer (27.9%, *n* = 306); (2) bile duct cancer (10.5%, *n* = 115); (3) colorectal cancer (10.2%, *n* = 112); (4) sarcoma (7.7%, *n* = 84); (5) gastric cancer (6.8%, *n* = 75); (6) lung cancer (5.6%, *n* = 61); (7) breast cancer (3.9%, *n* = 43); (8) prostate cancer (3.3%, *n* = 36); (9) esophagus cancer (2.9%, *n* = 32); and (10) ovarian cancer (2.9%, *n* = 32). The submitted gene panel included 900 (82.1%) FoundationOne CDx, 100 (9.1%) FoundationOne Liquid CDx, and 96 (8.8%) NCC Oncopanel cases (Figure 2B). The median TAT for FoundationOne CDx and NCC Oncopanel using tissue samples was 63 [28–182] days.

DNA was extracted and analyzed from the submitted specimens, and 117 cases (10.7% [117/1096]) were evaluated as qualified analysis. The top five types of cancer were pancreatic (29.1%, *n* = 34), gastric (13.7%, *n* = 16), bile duct (12.8%, *n* = 15), lung (7.7%, *n* = 9), and sarcoma (6.8%, *n* = 8). Of the qualified cases, 59 (50.4%) were surgical resection specimens, 52 (44.5%) were biopsy specimens, and 6 (5.1%) were blood specimens. Sixty (51.3%) patients had a TMB, and 70 (59.8%) patients had MSI that could not be determined.

### 3.2 Tumor mutation burden and distinct patterns of gene alteration co‐occurrence and mutual exclusivity

**FIGURE 3:**
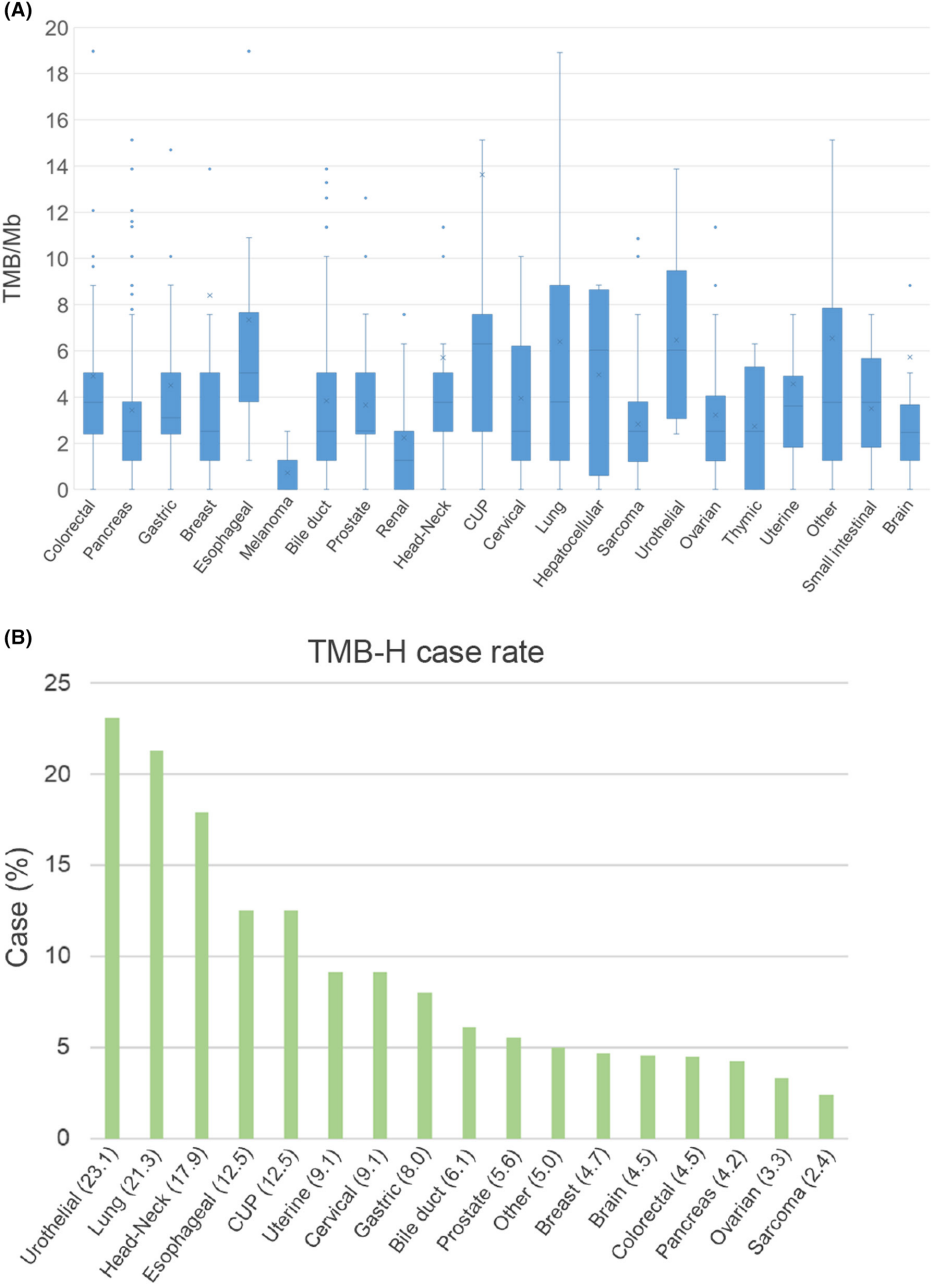
Box‐whisker plot of tumor mutation burden (TMB/Mb) per cancer type (A). TMB‐high (≥10/Mb) cases are shown in bar graphs for each cancer type (B). The figures in parentheses indicate the percentage of each cancer type among the total TMB‐high cases.

The results of the TMB are shown in Figure 3A. A median TMB of ≥5/Mb for cancer of unknown primary: 6.30 [range 0.00–136.38]/Mb, urothelial cancer: 6.03 [range 2.41–13.87]/Mb, hepatocellular cancer: 6.03 [range 0.00–8.85]/Mb, esophageal cancer: 5.05 [1.26–39.09]/Mb. The lowest TMB was for melanoma, 0 [0–2.52]/Mb. All of these melanomas were non sun‐exposed melanomas. Among all cases, 73 (6.7%) cases had TMB≥10/Mb. These cancer types and the percentage of cases with TMB≥10/Mb in each cancer type are shown in Figure 3B.

**FIGURE 4:**
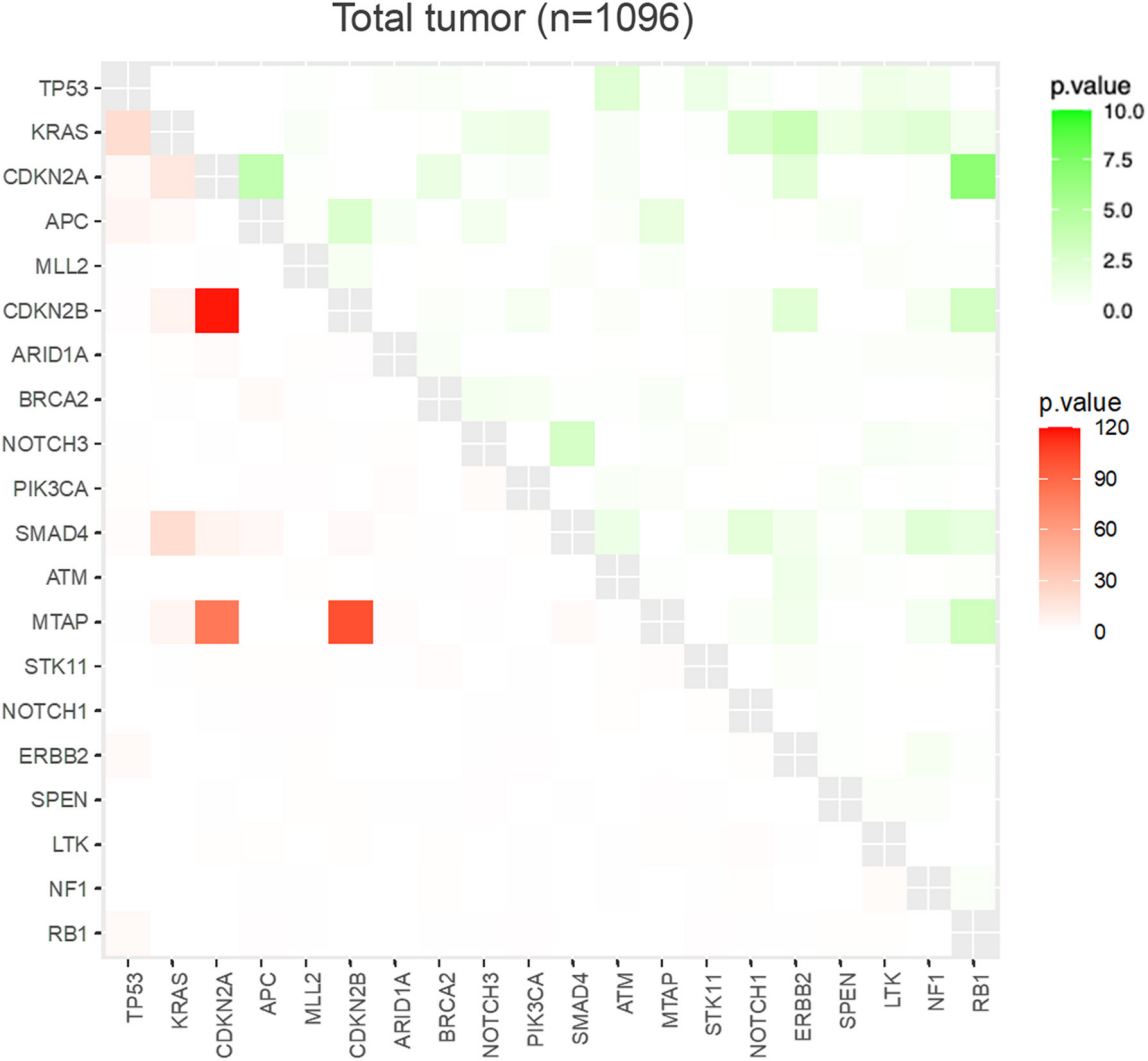
Co‐occurrence and mutual exclusivity of genetic alterations with significant interaction. The lower left region represents co‐occurrence (red), and the upper right region represents mutual exclusivity of genetic alterations (green).

The top 20 most frequent alterations detected for each cancer type were examined for their co‐occurrence and mutual exclusivity for the overall population (Figure 4). Co‐alterations of *CDKN2A*, *CDKN2B*, and *MTAP* was observed in the overall population.

### 3.3 Gene panel results led to genomically matched therapy in some cases

**FIGURE 5:**
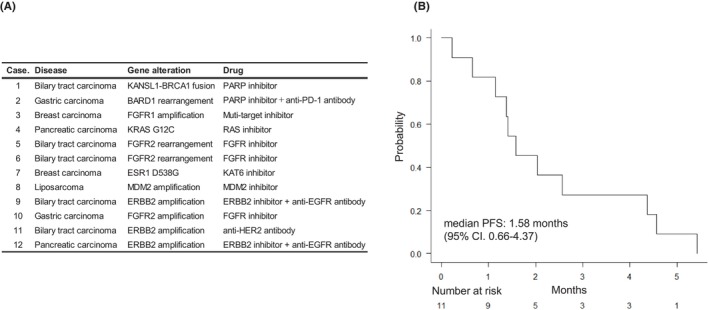
Genomically matched therapy in clinical trial. List of patients enrolled, cancer type, genetic alterations treated, and drugs introduced (A). Kaplan–Meier curve showing the progression free survival for the patients (B).

Based on the genetic alterations detected by the CGP panel results, there were 658 (60.0%) cases in which recommended clinical trials were presented. 550 of 658 were FoundationOne CDx (61.1% [550/900]), 46 FoundationOne Liquid CDx (46% [46/100]) and 62 NCC Oncopanel (64.6% [62/96]). Table 1 shows the recommended actionable gene alterations and their frequencies for each of the top seven cancer types (pancreas, bile duct, colorectal, sarcoma, gastric, lung and breast) in total and in the largest number of cases. There were 12 (1.1 [12/1096]%, 1.8 [12/658]%) cases that could be enrolled in the trial. Their cancer types, detected gene alterations, and trial treatments are shown in Figure 5A. The median progression free survival (PFS) of the trial treatment was 1.58 months (95% CI: 0.66–4.37) (Figure 5B). All successfully registered facilities were in the Kansai area.

**TABLE 1:** The recommended actionable genes and their frequencies for each of the top seven cancer types.

Total: *n* = 658 (60.0%)			Pancreas: *n* = 235 (76.8%)			Bile duct: *n* = 64 (55.7%)			Colorectal: n = 84 (75.0%)			Sarcoma: *n* = 40 (47.6%)			Gastric: n = 32 (42.7%)			Lung: *n* = 35 (57.3%)			Breast: *n* = 28 (65.1%)		
Actionable alterations		(%)	Actionable alterations		(%)	Actionable alterations		(%)	Actionable alterations		(%)	Actionable alterations		(%)	Actionable alterations		(%)	Actionable alterations		(%)	Actionable alterations		(%)
KRAS	301	35.0	KRAS	197	65.9	KRAS	17	20.0	KRAS	52	42.3	MDM2	20	34.5	ERBB2	10	21.3	EGFR	11	27.5	ERBB2	6	16.7
ERBB2	51	5.9	TP53	17	5.7	ERBB2	10	11.8	APC	36	29.3	CDK4	9	15.5	TP53	6	12.8	ERBB2	7	17.5	TP53	6	16.7
TMB high	50	5.8	CDKN2A	14	4.7	FGFR2	9	10.6	BRAF	6	4.9	KIT	4	6.9	KRAS	5	10.6	TMB high	6	15	FGFR1	5	13.9
TP53	48	5.6	BRCA1/2	13	4.3	PIK3CA	6	7.1	TP53	5	4.1	MTAP	4	6.9	FGFR2	4	8.5	KRAS	5	12.5	ESR	4	11.1
APC	37	4.3	MTAP	12	4.0	TMB high	6	7.1	ERBB2	4	3.3	NRAS	3	5.2	FGFR3	3	6.4	TP53	3	7.5	PIK3CA	4	11.1
MTAP	37	4.3	TMB high	11	3.7	IDH1	5	5.9	TMB high	4	3.3	PDGFRA	3	5.2	CDKN2A	3	6.4	ALK	2	5	FGFR2	2	5.6
BRCA1/2	33	3.8	ARID1A	4	1.3	BRCA1/2	4	4.7	ATM	4	3.3	CDKN2A	2	3.4	PIK3CA	3	6.4	others	6	15	others	9	25.0
CDKN2A	31	3.6	ERBB2	4	1.3	FGFR1	4	4.7	MSI	3	2.4	KDR	2	3.4	TMB high	3	6.4						
MDM2	30	3.5	ATM	3	1.0	MTAP	4	4.7	NRAS	3	2.4	TP53	2	3.4	ATM	2	4.3						
PIK3CA	23	2.7	BRAF	2	0.7	CDKN2A	3	3.5	MTAP	2	1.6	others	9	15.5	MSI	2	4.3						
FGFR2	19	2.2	MDM2	2	0.7	ATM	2	2.4	others	4	3.3				others	6	12.8						
ATM	17	2.0	MAP2K1	2	0.7	MDM2	2	2.4															
BRAF	16	1.9	MSI	2	0.7	SDHA	2	2.4															
FGFR1	15	1.7	others	16	5.4	TP53	2	2.4															
CDK4/6	13	1.5				others	9	10.6															
EGFR	12	1.4																					
MSI	10	1.2																					
FGFR3	9	1.0																					
CCND1	9	1.0																					
ARID1A	7	0.8																					
KIT	6	0.7																					
PTEN	6	0.7																					
MET	5	0.6																					
others	75	8.7																					

**FIGURE 6:**
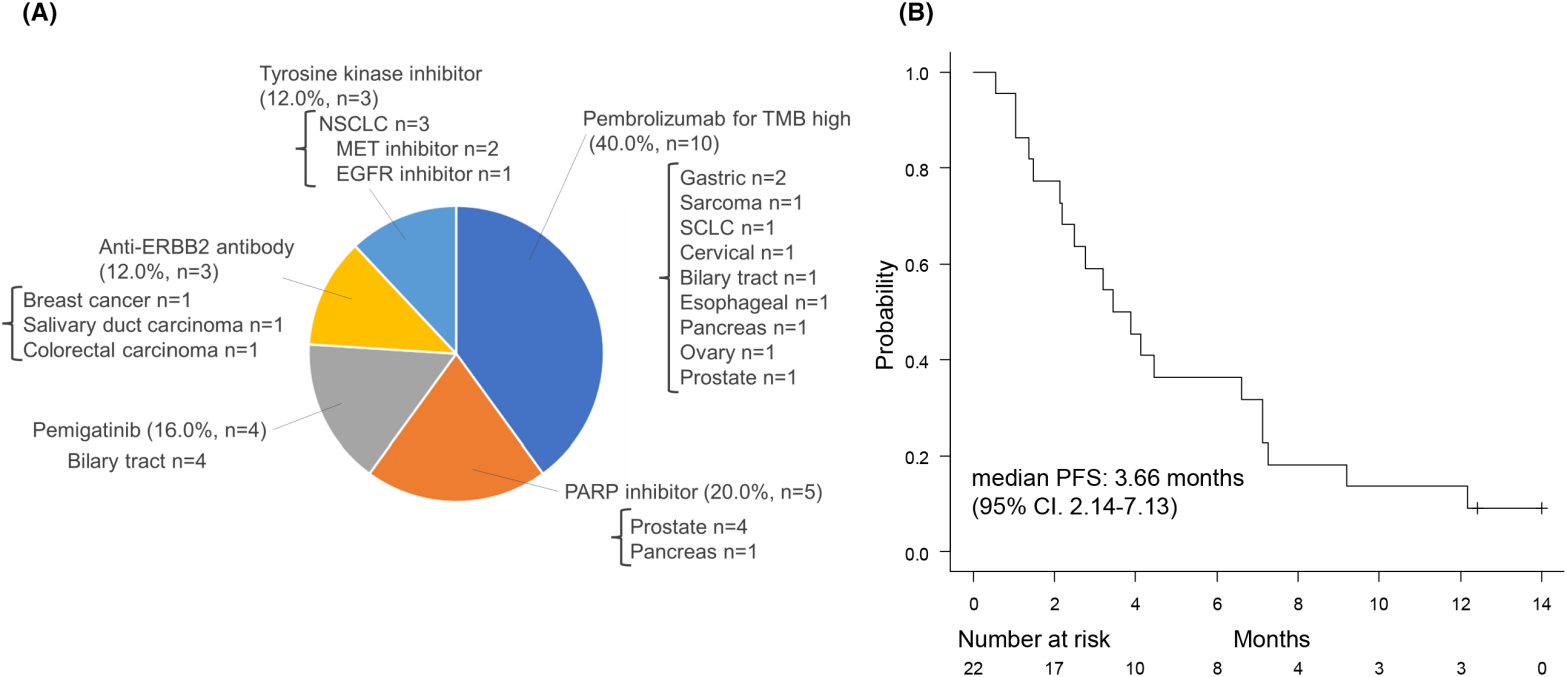
Genomically matched therapy under insurance coverage. List of drugs introduced, and cancer types covered (A). Kaplan–Meier curve showing progression free survival for the patients (B). EGFR, Epidermal growth factor receptor; ERBB2, Erb‐b2 receptor tyrosine kinase 2; MET, MET proto‐oncogene; NSCLC, Non‐small cell lung carcinoma; PARP, Poly(ADP‐ribose) polymerases; SCLC, Small cell lung carcinoma; TMB, Tumor mutation burden.

The genetic alterations detected by the CGP panel led to the introduction of therapies under insurance reimbursement in 2.3% [25/1096] and 3.8% [25/658] cases, and a list of therapeutics introduced and their respective cancer types is presented in Figure 6A. Ten (40.0%) patients received pembrolizumab for TMB‐high, five (20.0%) received PARP inhibitors, four (16.0%) received pemigatinib for FGFR2 fusion, three (12.0%) received anti‐ERBB2 antibody, and three (12.0%) received tyrosine kinase inhibitors. The median PFS in the 22 patients for whom treatment response could be evaluated was 3.66 months (95% CI: 2.14–7.13) (Figure 6B).

### 3.4 Secondary finding

**FIGURE 7:**
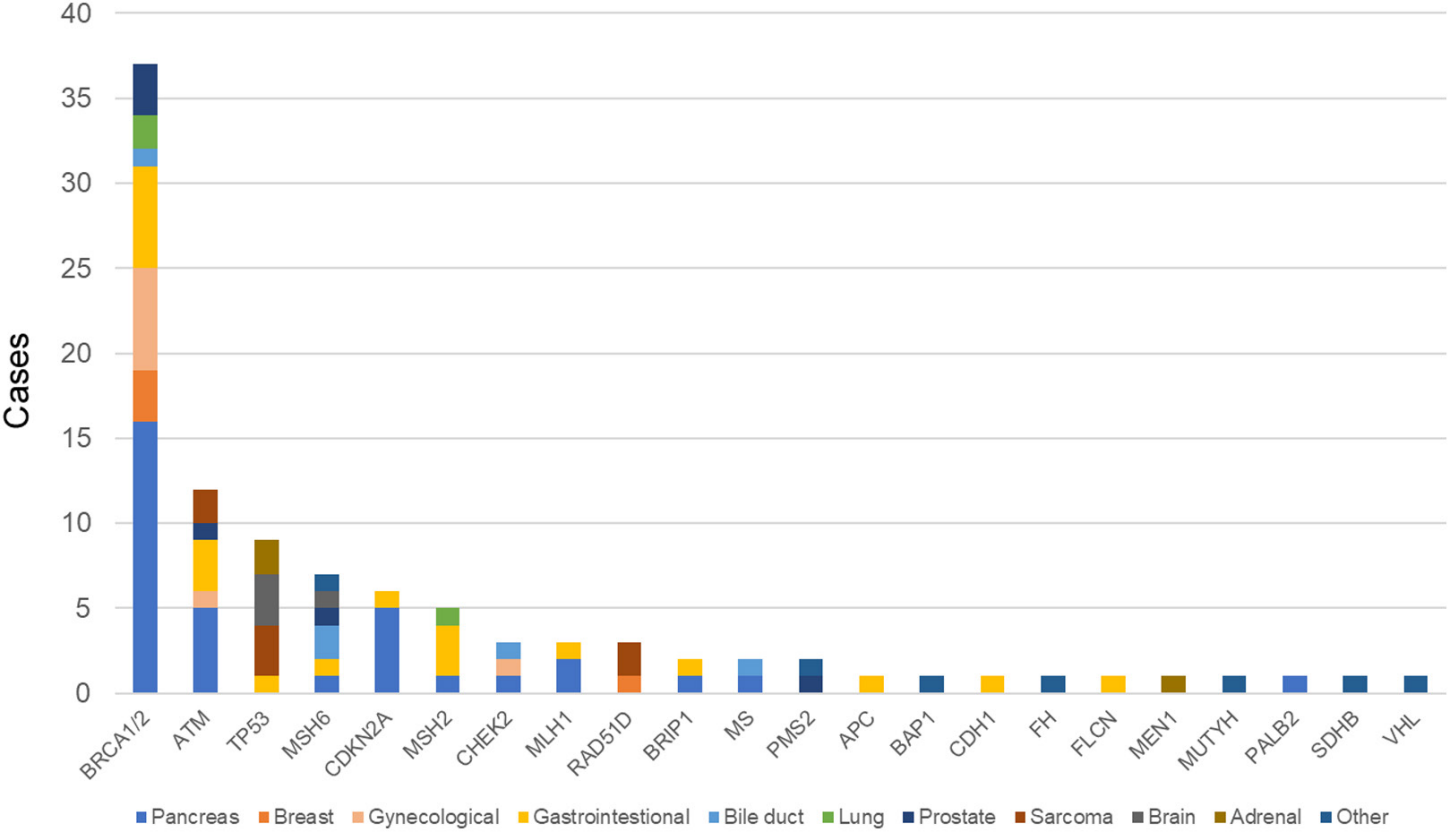
List of cases with secondary findings that were identified as possible pathologic germline variants. The number of cases for each genetic variant is indicated by a bar graph. The cancer type is color‐coded in the graph.

One hundred and one (9.2%) cases had mutations that were judged to be secondary mutations (Figure 7). The *BRCA*1/2 mutation was the most common (37 [36.6%] cases), followed by the *ATM* mutation (12 [11.9%] cases), *TP53* mutation (9 [8.9%] cases), *MSH6* mutation (7 [6.9%] cases), and *CDKN2A* mutation (6 [5.9%] cases). Of these, two cases of *BRCA2* and one case each of *ATM*, *CDKN2A*, and *CHEK2* mutations were confirmed as pathologic germline variants (PGVs) by the NCC panel. All 101 patients were recommended to receive genetic counseling; one patient died before receiving genetic counseling, and 57 (56.4%) cases received genetic counseling. Of the 43 patients who did not wish to receive genetic counseling, 26 patients themselves refused to receive counseling; for the remaining 17, no clear reason was given. 17 (29.8%) cases were tested for germline mutation as a result of genetic counseling. In 10 cases, mutations detected by tissue‐based panel were confirmed to be germline mutations. Pathogenic germline variants were identified in 1.3% of patients (15/1096).

## 4 DISCUSSION

Our study provides a comprehensive assessment of the utility and impact of CGP panels in clinical practice within a single institution in Japan. The unique contribution of this study lies in its real‐world approach, which reflects the clinical application of CGP within a diverse patient population encompassing various cancer types. To the best of our knowledge, no previous studies have evaluated the extent to which performing a CGP panel on patients with cancer who have completed standard treatment contributes to their life expectancy in a clinical practice setting.

In our study, genetic alterations identified by the CGP panel led to the inclusion of 12 patients in clinical trials for a drug under development, with a median mPFS of 1.58 months (95% CI: 0.66–4.37). Conversely, 22 patients received drugs covered by insurance, resulting in an mPFS of 3.66 months (95% CI: 2.14–7.13). A prospective study of the efficacy of genomically matched therapy in heavily pretreated patients with advanced solid tumors using the CGP panel showed an mPFS ranging from 2.3 to 2.7 months.^12^, ^13^, ^14^ While our institution's results were inferior to these reports for genomically matched therapy, they were superior when patients were linked to drugs covered by insurance. Notably, patients with a PFS of over 12 months were those who continued to receive pembrolizumab for TMB‐high.^29^

One of the contributing factors to the poor mPFS of under 2 months observed in our institution is the process of CGP panel analysis in clinical practice in Japan. This procedure requires expert panel review and it typically takes approximately 2 months for the test results to reach the patients at our institution. Further delays were incurred in the case of the 12 patients included in clinical trials outside our institute owing to the introduction of the trial drug, which involved referral to a clinical trial site and additional procedural steps.^30^ Regrettably, some patients were unable to receive the trial drug because their condition had deteriorated by the time they were enrolled and initiated treatment. Previous reports indicate that molecular tumor boards convene once a week and this may have contributed to the delayed initiation of treatment as the CGP panel results were interpreted.^12^ Although all 12 cases were enrolled in the Kansai area, challenges in enrolling patients at non‐Kansai area sites arose owing to variations in enrollment status at each site and other factors. Genomically matched therapy has made significant strides in the treatment of lung cancer among solid tumors by successfully identifying targetable driver alterations.^9^, ^31^, ^32^ However, the identification of such alterations in other cancer types remains unsatisfactory, necessitating reliance on clinical trials for genomically matched therapy. The expansion of such clinical trials and the development of remote clinical trial options are crucial steps in advancing genomic medicine towards curative outcomes.^33^ It is also advisable to consider revising the system to include CGP panel testing at the outset of treatment to prevent delays in clinical trial enrollment owing to disease progression. In the future, the use of artificial intelligence and the establishment of an automated workflow to identify pathogenic germline variants will make it possible to shorten the time required for expert panels.^34^

Unlike molecular targeted therapy, immunotherapy (exemplified by pembolizumab) exerts a long‐term antitumor effect referred to as the “long tail effect”.^35^, ^36^ In this study, two patients with TMB‐high positively responded to pembrolizumab for over 12 months. TMB‐high was observed across various cancer types in this study. In terms of long‐term efficacy, immunotherapy holds more promise than genomically matched therapy, which is still in the developmental phase. In fact, immunotherapy has gained widespread approval for use in solid tumors^37^ and is covered by insurance in Japan since December 2019 when the CGP panel was introduced. However, it has not been approved for major cancer types such as sarcoma, brain tumors, colorectal cancer, pancreatic cancer, and other rare cancer types, making CGP panel analysis essential to explore the possibility of introducing immunotherapy in these tumors. In 117 cases (10.7% [117/1096]), TMB measurement was not feasible owing to DNA quality deterioration, possibly attributed to inadequate specimen preservation. The establishment of a specimen management system, including fixation time in formalin and inhibition time for surgical specimens in anticipation of CGP panels is considered essential. In addition to TMB‐high, the future is expected to establish missense mutations in the polymerase epsilon (*POLE*) gene as a biomarker for immunotherapy across various cancer types.^38^ Consequently, the need for CGP panels in rare cancers and cancers for which immunotherapy has not been approved is projected to continue to rise.

The most common combination of gene alterations observed overall was a combination of three genes, *CDKN2A*, *2B*, and *MTAP*. The *CDKN2A* locus located on chromosome 9p21 encodes two tumor suppressor proteins: p16 and p14ARF.^39^ It is closely situated to a cluster of genes that include *CDKN2B* and *MTAP*.^40^ *MTAP* on chromosome 9p is often co‐deleted in human tumors, frequently leading to homozygous deletion owing to its proximity to *CDKN2A*. Approximately 10% of all human cancers exhibit frequent deletions of both *CDKN2A* and *MTAP*. Moreover, certain cancers, such as malignant peripheral nerve sheath tumors (MPNST), mesothelioma, glioblastoma, and pancreatic ductal adenocarcinoma display an even higher prevalence of this genetic co‐deletion.^41^ This may be partly owing to the fact that pancreatic cancer was the most common type of cancer included in this study (27.9%, *n* = 306).

The discovery of hereditary cancer predisposition has important implications.^42^, ^43^ It benefits at‐risk individuals with personalized clinical management and precision therapies, like PARP inhibitors for germline *BRCA* mutations.^44^ Identifying inherited risk can also lead to family cascade testing, providing benefits to others.^45^ Detecting germline variants through tumor CGP is clinically significant. The detection rate of pathologic germline variants based on the tumor‐only sequencing platform is approximately 7–9% in studies including all solid tumors.^46^, ^47^ In our study, 15 patients (1.3%) had PGV and 101 patients (9.2%) were identified as having possible PGV, but only 57 patients (56.4%) received genetic counseling. It is necessary to examine why these patients could not be connected to genetic counseling in the future. It is necessary to examine why the patients could not be connected to genetic counseling in the future. The identification of PGVs may lead to the prevention of cancer development in families, and efforts should be made to make PGV findings obtained from a tumor‐only sequencing platform transferable to clinical practice despite the existence of a surveillance system.

Several limitations exist in this study. First, it is a single‐center, retrospective study, and there are biases such as the type of cancer enrolled. In fact, this study includes a large number of pancreatic cancer patients and does not adequately assess the efficacy of the CGP panel across cancer types. Therefore, large‐scale prospective integrated studies are required in the future. Second, the effectiveness of genomically matched therapy depends on how vigorously each case's attending physician enrolls the patient in the recommended trial; therefore, it is possible that there are cases that could not be enrolled in eligible trials. The effect of genomically matched therapy by the CGP panel may have been underestimated. Third, this study was conducted in the Kansai area of Japan which may be affected by the lack of opportunities for clinical trials owing to regional disparities, which may improve with the development of remote clinical trials.

## 5 CONCLUSION

Although the results of the introduction of genomically matched therapy based on the CGP panel at our institution were unsatisfactory, the CGP panel results have led to immunotherapy for TMB‐high for the first time in some cases, and long‐term antitumor effects were confirmed in some patients. In the future, it will be necessary to establish a cancer care system that fully utilizes the results of the CGP panel, with the aim of expanding clinical trials and enhancing and expanding care for PGV.

## AUTHOR CONTRIBUTIONS

**Kei Kunimasa:** Conceptualization (lead); data curation (lead); formal analysis (lead); investigation (lead); methodology (lead); project administration (lead); resources (lead); writing – original draft (lead); writing – review and editing (supporting). **Naotoshi Sugimoto:** Conceptualization (lead); data curation (lead); formal analysis (lead); investigation (lead); resources (lead); supervision (lead); writing – review and editing (lead). **Tomoyuki Yamasaki:** Formal analysis (supporting); investigation (supporting); resources (supporting). **Yoji Kukita:** Formal analysis (lead); investigation (supporting); resources (lead); software (lead). **Fumie Fujisawa:** Formal analysis (supporting); investigation (supporting); resources (supporting); writing – review and editing (supporting). **Tazuko Inoue:** Investigation (supporting); resources (supporting). **Yuko Yamaguchi:** Data curation (lead); resources (lead). **Mitsuko Kitasaka:** Methodology (supporting); resources (supporting). **Daisuke Sakai:** Formal analysis (supporting); investigation (supporting); resources (supporting). **Keiichiro Honma:** Formal analysis (supporting); investigation (supporting); resources (lead); supervision (supporting); writing – review and editing (supporting). **Toru Wakamatsu:** Formal analysis (supporting); investigation (supporting); resources (supporting). **Sachiko Yamamoto:** Formal analysis (supporting); investigation (supporting); resources (supporting). **Takuji Hayashi:** Formal analysis (supporting); investigation (supporting); resources (supporting). **Seiji Mabuchi:** Formal analysis (supporting); investigation (supporting); resources (supporting). **Jun Okuno:** Formal analysis (supporting); investigation (supporting); resources (supporting). **Takahisa Kawamura:** Formal analysis (supporting); investigation (supporting); resources (supporting). **Yugo Kai:** Formal analysis (supporting); investigation (supporting); resources (supporting). **Makiko Urabe:** Formal analysis (supporting); investigation (supporting); resources (supporting). **Kazuo Nishimura:** Conceptualization (supporting); investigation (supporting); supervision (lead); writing – review and editing (lead).

## FUNDING INFORMATION

The authors did not receive support from any organization for the submitted work.

## CONFLICT OF INTEREST STATEMENT

Dr. Kunimasa reports honoraria for lecture from Chugai Pharma and Dr. Nishimura reports honoraria for lectures from Astellas, AstraZeneca, MSD, Janssen, Merck Biopharma and Bayer. The other authors have no conflict of interest.

## ETHICS STATEMENT

The study was approved by the ethics committee of the Osaka International Cancer Center (#23199).

## THE USE OF GENERATIVE AI AND AI‐ASSISTED TECHNOLOGIES

During the preparation of this study, the authors did not use AI or AI‐assisted technologies. An external contractor performed English proofreading and grammar checks.

## Data Availability

The data that support the findings of this study are not publicly available due to their containing information that could compromise the privacy of research participants but are available from the corresponding author (Kei Kunimasa, kei.kunimasa@oici.jp) upon reasonable request. Further enquiries can be directed to the corresponding author.

## References

[1] Wahida A , Buschhorn L , Fröhling S , et al. The coming decade in precision oncology: six riddles. Nat Rev Cancer. 2023;23:43‐54. 36434139 10.1038/s41568-022-00529-3

[2] Yoshida T , Yatabe Y , Kato K , et al. The evolution of cancer genomic medicine in Japan and the role of the National Cancer Center Japan. Cancer Biol Med. 2023;21:29‐44. 37133223 10.20892/j.issn.2095-3941.2023.0036 PMC10875288

[3] Ebi H , Bando H . Precision oncology and the universal health coverage system in Japan. JCO Precis Oncologia. 2019;3:PO.19.00291. 10.1200/PO.19.00291 PMC7446489 32923862

[4] Fulton‐Ward T , Middleton G . The impact of genomic context on outcomes of solid cancer patients treated with genotype‐matched targeted therapies: a comprehensive review. Ann Oncol. 2023;34:1113‐1130. 37875224 10.1016/j.annonc.2023.10.124

[5] Radovich M , Kiel PJ , Nance SM , et al. Clinical benefit of a precision medicine based approach for guiding treatment of refractory cancers. Oncotarget. 2016;7:56491‐56500. 27447854 10.18632/oncotarget.10606 PMC5302930

[6] Olsen S , Liao J , Hayashi H . Real‐world clinical outcomes after genomic profiling of circulating tumor DNA in patients with previously treated advanced non‐small cell lung cancer. Curr Oncol. 2022;29:4811‐4826. 35877242 10.3390/curroncol29070382 PMC9318660

[7] Lemmon CA , Zhou J , Hobbs B , Pennell NA . Modeling costs and life‐years gained by population‐wide next‐generation sequencing or single‐gene testing in nonsquamous non‐small‐cell lung cancer in the United States. JCO Precis Oncol. 2023;7:e2200294. 36634300 10.1200/PO.22.00294 PMC9928881

[8] Arriola E , Bernabé R , Campelo RG , et al. Cost‐effectiveness of next‐generation sequencing versus single‐gene testing for the molecular diagnosis of patients with metastatic non‐small‐cell lung cancer from the perspective of Spanish reference centers. JCO Precis Oncol. 2023;7:e2200546. 36862967 10.1200/PO.22.00546 PMC10309530

[9] Middleton G , Fletcher P , Popat S , et al. The National Lung Matrix Trial of personalized therapy in lung cancer. Nature. 2020;583:807‐812. 32669708 10.1038/s41586-020-2481-8 PMC7116732

[10] Koroukian SM , Booker BD , Vu L , et al. Receipt of targeted therapy and survival outcomes in patients with metastatic colorectal cancer. JAMA Netw Open. 2023;6:e2250030. 36656585 10.1001/jamanetworkopen.2022.50030 PMC9857024

[11] Andre F , Filleron T , Kamal M , et al. Genomics to select treatment for patients with metastatic breast cancer. Nature. 2022;610:343‐348. 36071165 10.1038/s41586-022-05068-3

[12] Le Tourneau C , Delord JP , Gonçalves A , et al. Molecularly targeted therapy based on tumour molecular profiling versus conventional therapy for advanced cancer (SHIVA): a multicentre, open‐label, proof‐of‐concept, randomised, controlled phase 2 trial. Lancet Oncol. 2015;16:1324‐1334. 26342236 10.1016/S1470-2045(15)00188-6

[13] Massard C , Michiels S , Ferté C , et al. High‐throughput genomics and clinical outcome in hard‐to‐treat advanced cancers: results of the MOSCATO 01 trial. Cancer Discov. 2017;7:586‐595. 28365644 10.1158/2159-8290.CD-16-1396

[14] Bertucci F , Gonçalves A , Guille A , et al. Prospective high‐throughput genome profiling of advanced cancers: results of the PERMED‐01 clinical trial. Genome Med. 2021;13:87. 34006291 10.1186/s13073-021-00897-9 PMC8132379

[15] Hayeems RZ , Dimmock D , Bick D , et al. Clinical utility of genomic sequencing: a measurement toolkit. NPJ Genom Med. 2020;5:56. 33319814 10.1038/s41525-020-00164-7 PMC7738524

[16] Kotze MJ , Lückhoff HK , Peeters AV , et al. Genomic medicine and risk prediction across the disease spectrum. Crit Rev Clin Lab Sci. 2015;52:120‐137. 25597499 10.3109/10408363.2014.997930

[17] Tögel L , Schubart C , Lettmaier S , et al. Determinants affecting the clinical implementation of a molecularly informed molecular tumor board recommendation: experience from a tertiary cancer center. Cancers (Basel). 2023;15:5892. 38136436 10.3390/cancers15245892 PMC10741918

[18] Ishimaru S , Shimoi T , Sunami K , et al. Platform trial for off‐label oncology drugs using comprehensive genomic profiling under the universal public healthcare system: the BELIEVE trial. Int J Clin Oncol. 2023;29:89‐95. 38112833 10.1007/s10147-023-02439-2 PMC10808137

[19] Mosteiro M , Azuara D , Villatoro S , et al. Molecular profiling and feasibility using a comprehensive hybrid capture panel on a consecutive series of non‐small‐cell lung cancer patients from a single centre. ESMO Open. 2023;8:102197. 38070435 10.1016/j.esmoop.2023.102197 PMC10774954

[20] Kunimasa K , Sugimoto N , Kawamura T , et al. Clinical application of comprehensive genomic profiling panel to thoracic malignancies: a single‐center retrospective study. Thorac Cancer. 2022;13:2970‐2977. 36100256 10.1111/1759-7714.14643 PMC9626350

[21] Yamai T , Ikezawa K , Sugimoto N , et al. Utility of comprehensive genomic profiling tests for patients with incurable pancreatic cancer in clinical practice. Cancers (Basel). 2023;15:15. 10.3390/cancers15030970 PMC9913675 36765927

[22] Sunami K , Naito Y , Aimono E , et al. The initial assessment of expert panel performance in core hospitals for cancer genomic medicine in Japan. Int J Clin Oncol. 2021;26:443‐449. 33385275 10.1007/s10147-020-01844-1 PMC7895780

[23] Mukai Y , Ueno H . Establishment and implementation of cancer genomic medicine in Japan. Cancer Sci. 2021;112:970‐977. 33289217 10.1111/cas.14754 PMC7935799

[24] Li MM , Datto M , Duncavage EJ , et al. Standards and guidelines for the interpretation and reporting of sequence variants in cancer: a joint consensus recommendation of the Association for Molecular Pathology, American Society of Clinical Oncology, and College of American Pathologists. J Mol Diagn. 2017;19:4‐23. 27993330 10.1016/j.jmoldx.2016.10.002 PMC5707196

[25] Sunami K , Ichikawa H , Kubo T , et al. Feasibility and utility of a panel testing for 114 cancer‐associated genes in a clinical setting: a hospital‐based study. Cancer Sci. 2019;110:1480‐1490. 30742731 10.1111/cas.13969 PMC6447843

[26] Miller DT , Lee K , Gordon AS , et al. Recommendations for reporting of secondary findings in clinical exome and genome sequencing, 2021 update: a policy statement of the American College of Medical Genetics and Genomics (ACMG). Genet Med. 2021;23:1391‐1398. 34012069 10.1038/s41436-021-01171-4 PMC12175738

[27] Kanda Y . Investigation of the freely available easy‐to‐use software ‘EZR’ for medical statistics. Bone Marrow Transplant. 2013;48:452‐458. 23208313 10.1038/bmt.2012.244 PMC3590441

[28] Lengel HB , Mastrogiacomo B , Connolly JG , et al. Genomic mapping of metastatic organotropism in lung adenocarcinoma. Cancer Cell. 2023;41:970‐985.e3. 37084736 10.1016/j.ccell.2023.03.018 PMC10391526

[29] Cristescu R , Mogg R , Ayers M , et al. Pan‐tumor genomic biomarkers for PD‐1 checkpoint blockade‐based immunotherapy. Science. 2018;362:eaar3593. 30309915 10.1126/science.aar3593 PMC6718162

[30] Heitjan DF , Ge Z , Ying GS . Real‐time prediction of clinical trial enrollment and event counts: a review. Contemp Clin Trials. 2015;45:26‐33. 26188165 10.1016/j.cct.2015.07.010

[31] Mosele F , Remon J , Mateo J , et al. Recommendations for the use of next‐generation sequencing (NGS) for patients with metastatic cancers: a report from the ESMO precision medicine working group. Ann Oncol. 2020;31:1491‐1505. 32853681 10.1016/j.annonc.2020.07.014

[32] Ettinger DS , Wood DE , Aisner DL , et al. Non‐small cell lung cancer, version 3.2022, NCCN clinical practice guidelines in oncology. J Natl Compr Cancer Netw. 2022;20:497‐530. 10.6004/jnccn.2022.0025 35545176

[33] Brown NA , Elenitoba‐Johnson KSJ . Enabling precision oncology through precision diagnostics. Annu Rev Pathol. 2020;15:97‐121. 31977297 10.1146/annurev-pathmechdis-012418-012735

[34] Sunami K , Naito Y , Saigusa Y , et al. A learning program for treatment recommendations by molecular tumor boards and artificial intelligence. JAMA Oncologia. 2023;10:95. 10.1001/jamaoncol.2023.5120 PMC10690580 38032680

[35] Wolchok JD , Chiarion‐Sileni V , Gonzalez R , et al. Long‐term outcomes with Nivolumab plus Ipilimumab or Nivolumab alone versus Ipilimumab in patients with advanced melanoma. J Clin Oncol. 2022;40:127‐137. 34818112 10.1200/JCO.21.02229 PMC8718224

[36] Reck M , Rodríguez‐Abreu D , Robinson AG , et al. Five‐year outcomes with Pembrolizumab versus chemotherapy for metastatic non‐small‐cell lung cancer with PD‐L1 tumor proportion score ≥ 50. J Clin Oncol. 2021;39:2339‐2349. 33872070 10.1200/JCO.21.00174 PMC8280089

[37] Twomey JD , Zhang B . Cancer immunotherapy update: FDA‐approved checkpoint inhibitors and companion diagnostics. AAPS J. 2021;23:39. 33677681 10.1208/s12248-021-00574-0 PMC7937597

[38] Rousseau B , Bieche I , Pasmant E , et al. PD‐1 blockade in solid tumors with defects in polymerase epsilon. Cancer Discov. 2022;12:1435‐1448. 35398880 10.1158/2159-8290.CD-21-0521 PMC9167784

[39] Gu F , Pfeiffer RM , Bhattacharjee S , et al. Common genetic variants in the 9p21 region and their associations with multiple tumours. Br J Cancer. 2013;108:1378‐1386. 23361049 10.1038/bjc.2013.7 PMC3619272

[40] Cerami E , Gao J , Dogrusoz U , et al. The cBio cancer genomics portal: an open platform for exploring multidimensional cancer genomics data. Cancer Discov. 2012;2:401‐404. 22588877 10.1158/2159-8290.CD-12-0095 PMC3956037

[41] Mulvaney KM . Early clinical success of MTA‐cooperative PRMT5 inhibitors for the treatment of CDKN2A/MTAP‐deleted cancers. Cancer Discov. 2023;13:2310‐2312. 37909092 10.1158/2159-8290.CD-23-0951

[42] Meric‐Bernstam F , Brusco L , Daniels M , et al. Incidental germline variants in 1000 advanced cancers on a prospective somatic genomic profiling protocol. Ann Oncol. 2016;27:795‐800. 26787237 10.1093/annonc/mdw018 PMC4843184

[43] Schrader KA , Cheng DT , Joseph V , et al. Germline variants in targeted tumor sequencing using matched Normal DNA. JAMA Oncol. 2016;2:104‐111. 26556299 10.1001/jamaoncol.2015.5208 PMC5477989

[44] Maiorano BA , Conteduca V , Catalano M , et al. Personalized medicine for metastatic prostate cancer: the paradigm of PARP inhibitors. Crit Rev Oncol Hematol. 2023;192:104157. 37863403 10.1016/j.critrevonc.2023.104157

[45] Mandelker D , Zhang L , Kemel Y , et al. Mutation detection in patients with advanced cancer by universal sequencing of cancer‐related genes in tumor and Normal DNA vs guideline‐based germline testing. JAMA. 2017;318:825‐835. 28873162 10.1001/jama.2017.11137 PMC5611881

[46] Kage H , Shinozaki‐Ushiku A , Ishigaki K , et al. Clinical utility of Todai OncoPanel in the setting of approved comprehensive cancer genomic profiling tests in Japan. Cancer Sci. 2023;114:1710‐1717. 36601953 10.1111/cas.15717 PMC10067384

[47] Tung N , Dougherty KC , Gatof ES , et al. Potential pathogenic germline variant reporting from tumor comprehensive genomic profiling complements classic approaches to germline testing. NPJ Precis Oncol. 2023;7:76. 37568048 10.1038/s41698-023-00429-1 PMC10421918

